# Beech Fructification and Bank Vole Population Dynamics - Combined Analyses of Promoters of Human Puumala Virus Infections in Germany

**DOI:** 10.1371/journal.pone.0134124

**Published:** 2015-07-27

**Authors:** Daniela Reil, Christian Imholt, Jana Anja Eccard, Jens Jacob

**Affiliations:** 1 Julius Kühn-Institute, Federal Research Centre for Cultivated Plants, Institute for Plant Protection in Horticulture and Forests, Vertebrate Research, Muenster, Germany; 2 University of Potsdam, Institute of Biochemistry and Biology, Animal Ecology, Potsdam, Germany; Linneaus University, SWEDEN

## Abstract

The transmission of wildlife zoonoses to humans depends, amongst others, on complex interactions of host population ecology and pathogen dynamics within host populations. In Europe, the Puumala virus (PUUV) causes nephropathia epidemica in humans. In this study we investigated complex interrelations within the epidemic system of PUUV and its rodent host, the bank vole (*Myodes glareolus*). We suggest that beech fructification and bank vole abundance are both decisive factors affecting human PUUV infections. While rodent host dynamics are expected to be directly linked to human PUUV infections, beech fructification is a rather indirect predictor by serving as food source for PUUV rodent hosts. Furthermore, we examined the dependence of bank vole abundance on beech fructification. We analysed a 12-year (2001-2012) time series of the parameters: beech fructification (as food resource for the PUUV host), bank vole abundance and human incidences from 7 Federal States of Germany. For the first time, we could show the direct interrelation between these three parameters involved in human PUUV epidemics and we were able to demonstrate on a large scale that human PUUV infections are highly correlated with bank vole abundance in the present year, as well as beech fructification in the previous year. By using beech fructification and bank vole abundance as predictors in one model we significantly improved the degree of explanation of human PUUV incidence. Federal State was included as random factor because human PUUV incidence varies considerably among states. Surprisingly, the effect of rodent abundance on human PUUV infections is less strong compared to the indirect effect of beech fructification. Our findings are useful to facilitate the development of predictive models for host population dynamics and the related PUUV infection risk for humans and can be used for plant protection and human health protection purposes.

## Introduction

Numerous wildlife zoonoses (majority of emerging pathogens; [1]) are transmitted to humans by rodents, because rodents are an abundant and species-rich group of mammals that carry a wide variety of pathogens [2], such as leptospirosis, murine typhus, influenza, plague, trypanosomiasis, salmonellosis and toxoplasmosis [2, 3]. Rodents have a quick alternation of generations and if environmental conditions are favourable a high reproductive success. They can reach high densities quickly, which faciliates the transmission of pathogens [4, 5].

Habitat structure and food availability have an impact on host population dynamics, which in turn are linked to human infection risk with zoonotic pathogens. For example the only environmental parameter linked to an increased infection rate with tick-borne Lyme disease in humans is the occurrence of woodland [6]. Other studies revealed the dependence of human infection risk with vector-borne pathogens on the preceding rodent host abundance and the abundance of acorns (food resource for key host) two years prior to the infection risk [7, 8]. However, there are several factors affecting population dynamics of rodents (reviewed in [9]) and subsequently the transmission of zoonoses to humans [10, 11], including hantavirus infections.

The hantavirus is a zoonotic pathogen of global interest [12]. Hantaviruses are worldwide distributed and each species has a specific rodent reservoir species or group of closely related rodent species (e.g. Seoul virus—Norway rat (*Rattus norvegicus*), Sin Nombre virus—*Peromyscus* spec.). The Old and New World hantaviruses cause either hemorrhagic fever with renal syndrom (HFRS) or hantavirus pulmonary syndrom (HPS). Usually, hantaviruses are carried by rodents, but recently also other hosts were discovered such as shrews, moles, and bats [13–15]. In Europe, the most widely distributed hantavirus species is the Puumala virus (PUUV), first described in Finland in 1980 [16], where the disease is very common [17]. An infection with PUUV in Europe causes a mild form of HFRS, called *nephropathia epidemica* (NE). PUUV in Europe is predominantly transmitted by bank voles (*Myodes glareolus*).

Hantaviruses are single-stranded, three-segmented, negative-sense RNA genomes coding for nucleocapsid proteins [18] and cause persistent infections in their rodent host species, which remain usually apathogenic [19]. However, reproduction and winter survival can be negatively affected [20, 21]. In contrast to asymptomatic infections in rodent hosts, human infections with hantavirus can lead from mild to severe illness after an incubation period of two to four weeks [22, 23]—as the above-mentioned NE.

In Germany, the awareness for hantavirus infections in humans rose in the 1980s, when NE was clinically described for the first time in 1984 during a Belgian military exercise in North Rhine-Westphalia [24], and the first case of acute kidney failure caused by Hantaan virus (another hantavirus species) was reported in Western Germany in 1985 [25]. The first NE outbreak in Germany was recorded in 1990 with 24 positive cases in only 2 weeks in Baden-Wuerttemberg during an American military exercise [26]. Since 2001, hantavirus infection is a notifiable disease that has to be reported to the Public Health Institute for Germany (Robert-Koch-Institute, RKI), and since then the number of reported cases in outbreak years has steadily increased (RKI, SurvStat, http://www3.rki.de/SurvStat).

The reservoir species for PUUV, the bank vole, is widely spread throughout Europe. It inhabits boreal forests in Northern Europe but also temperate forests in Western and Central Europe, dominated by deciduous broad-leaved trees, e.g. beech (*Fagus sylvatica*) or oak (*Quercus robur*) [27]. Vole population dynamics in Fennoscandia seem to be mainly predator driven [28] (though other explanations have been discussed [29]), while in temperate Europe they may be resource driven since masting of tree seeds precedes bank vole outbreaks [30, 31]. In Central Europe, bank voles prefer deciduous tree seeds [31, 32]. They prefer beech seeds to acorns and hazel nuts (*Corylus avellana*) [33]. In Germany, beech is an important broad-leaved tree species, covering 14.8% of the forest area [34].

In the northern boreal zone human infections with PUUV can be predicted by rodent host population cycles even without consideration of the prevalence of PUUV in the reservoir [35, 36]. In Central Europe, beech mast precedes bank vole outbreaks [30, 31] and outbreaks of NE cases in humans [37, 38]. Beech mast and the following NE outbreaks seem to be triggered by warm autumn and summer temperatures in the 1–2 years before NE peaks [37, 38]. Peaks of human NE cases in Central Europe usually occur in early summer associated with high bank vole abundances in the same year [37]. In Northern Europe, NE cases normally peak in late autumn-early winter. If high spring or summer NE incidences occur, they are more likely to be linked to higher bank vole densities in the previous fall [35, 36, 39]. On a regional scale in Southern Germany, Piechotowski, Brockmann [40] propose that human hantavirus infections are dependent on several interacting factors such as rodent host density, seasonal climate changes (mild winters, early spring), and food conditions, as well as human exposure. However, in a recent review it was pointed out that there is a lack of systematic and long-term studies in several European countries including Germany [41].

In this study, we investigated two parameters potentially impacting human PUUV incidence in Germany: 1) fructification of common beech as food source for bank voles, and 2) abundance of bank voles as PUUV host species. Previously, beech mast and human NE cases [27, 37], beech mast and bank vole dynamics [42], or bank vole dynamics and NE cases [35, 36] have been linked. We here present for the first time a combined analyses of all three factors likely to be involved in human PUUV epidemics using well replicated time series collected over 12 years on large spatial scale throughout Germany.

We investigated a possible connection of human PUUV infections to host population dynamics in Germany to find out if the rising number of human infections is not simply a consequence of increased awareness towards the disease. Further we analysed the relative importance of beech fructification for bank vole population dynamics and human NE incidences. We hypothesised that:

1. the incidence of human PUUV infections is directly dependent on bank vole abundance,
2. beech fructification affects bank vole abundance positively in the following year, and
3. human PUUV incidence is more strongly linked to rodent abundance than to beech fructification.

## Materials and Methods

Time series of beech fructification, PUUV rodent host abundance, and human PUUV infections in Germany were obtained from public authorities including Forest State Agencies and Federal Research Institutes. Time series used for analyses covered 12 years (2001–2012) because recording of human PUUV infections started in 2001. Time series originated from 7 (rodent abundance) or 11 Federal States (beech fructification) corresponding to an area of 227,000–337,000 km^2^.

### Beech fructification

Data on beech fructification were either published in state forest status reports or provided by Forest Authorities. Information was available for 11 Federal States from 2000–2012 where beech fructification was estimated between July and August each year as the percentage of fruiting beech trees, classified in absent, scarce, common, and abundant fructification (Table 1, [43]). Data were recorded at multiple locations per state as the cumulative number of trees in each class. Beech trees older than 49 years, but usually older than 60 years, were included in this estimation, because younger trees do not develop fruits extensively. Fructification was considered masting when there were more than one third of beech trees with common and/or abundant fructification.

**Table 1:** Classification of tree fructification [43].

Class	Description
absent	fructification is absent or inconsiderable; even reasonably lengthy observation of the crown with binoculars yields no signs of fruiting
scarce	sporadic occurrence of fruiting, not noticeable at first sight; it must be looked for on purpose with binoculars
common	fructification can be observed with the naked eye; the appearance of the tree is influenced but not dominated by fructification
abundant	fructification is obvious and immediately meets the eye; dominates the tree's appearance
mast	more than one third of beech trees with common and/or abundant fructification

Further, the proportion of beech forest per state was calculated from the size of beech forests per state (source: *National Forest Inventory* in 2002, http://www.bundeswaldinventur.de) and the size of the respective Federal State (source: *Statistisches Bundesamt*, *Gemeindeverzeichnis GV-ISys*, https://www.destatis.de, data status: 31-Dec-2012).

### Bank vole abundance

Information about bank vole abundance was available for 7 Federal States. Bank vole time series were provided by Forest Authorities, which monitor forest rodents for plant protection purposes. Data were collected annually in autumn at multiple locations by the Northwest German Forest Research Station and the Lower Saxony State Office for Consumer Protection and Food Safety (Lower Saxony 2001–2012; Hesse 2006–2012), the Brandenburg Forestry State Agency (Brandenburg 2001–2011), the State Forest Mecklenburg-Western Pomerania (Mecklenburg-Western Pomerania 2001–2006), the Public Enterprise Sachsenforst (Saxony 2001–2012), ThüringenForst (Thuringia 2001–2012), and the Bavarian State Institute of Forestry (Bavaria 2008–2010). Time series thus covered 3–12 consecutive years (N = 63).

In standardized trapping sessions, mostly conducted on afforestation plots, the number of trapped individuals per 100 effective trap nights (TN) using snap traps was estimated. Normally, 100 snap traps were set for one night or 50 traps for two consecutive nights. After 24 hours, the number of sprung traps was subtracted from the number of set traps to calculate the number of effective traps. The number of individuals of a particular species caught was converted to an adjusted trap success of individuals per 100 effective trap nights (ind/100TN; bank vole abundance).

### Human PUUV infections

In Germany, 87% of human hantavirus infections are due to PUUV and 11% are undifferentiated, hence only 2% are diagnosed to be caused by other hantavirus species, such as Dobrava-Belgrade virus (DOBV) (http://www3.rki.de/SurvStat). Human PUUV infection data were retrieved from RKI, where infections are recorded according to residence of the patient. The number of human PUUV cases (Table 2) in relation to the number of inhabitants per year and per Federal State (human PUUV incidence) were included in the analyses.

**Table 2:** Number of notified human PUUV infections per Federal State in Germany from 2001–2012.

Federal State	2001	2002	2003	2004	2005	2006	2007	2008	2009	2010	2011	2012	Total	
Baden-Wuerttemberg	36	140	55	109	105	17	1,076	70	75	946	114	1,485	4,258	**56.1%**
Bavaria	17	9	15	58	40	11	289	40	20	415	38	379	1,367	**18.0%**
Berlin	0	1	0	0	2	0	1	1	0	3	0	0	8	**0.1%**
Brandenburg	0	0	0	0	1	0	1	0	0	1	1	2	6	**0.1%**
Bremen	1	0	0	1	0	0	0	0	0	1	0	2	5	**0.1%**
Hamburg	0	0	0	0	0	0	2	0	1	0	1	3	7	**0.1%**
Hesse	10	4	11	5	33	2	25	10	3	162	5	89	363	**4.8%**
Mecklenburg-Western Pomerania	0	0	0	0	0	0	1	0	1	2	0	0	4	**0.1%**
Loxer Saxony	6	3	2	9	60	6	86	14	13	116	13	118	452	**6.0%**
North Rhine-Westphalia	36	14	17	26	120	18	122	60	28	139	53	164	813	**10.7%**
Rhineland-Palatinate	2	0	2	3	10	0	8	4	1	16	3	63	113	**1.5%**
Saarland	0	0	0	0	0	0	2	0	0	1	0	8	11	**0.1%**
Saxony	0	0	0	0	0	1	3	0	0	3	0	7	15	**0.2%**
Saxony-Anhalt	0	0	1	0	0	0	0	0	1	5	0	6	13	**0.2%**
Schleswig-Holstein	0	0	0	1	3	2	3	0	0	5	2	5	21	**0.3%**
Thuringia	2	1	3	0	13	0	6	6	0	58	3	38	133	**1.8%**
**Total**	**110**	**172**	**106**	**212**	**387**	**57**	**1,625**	**205**	**143**	**1,873**	**233**	**2,369**	**7,589**	**100.0%**

Source: Robert Koch-Institute, SurvStat, http://www3.rki.de/SurvStat, data status: 06/27/2014.

### Statistical analyses

The influences of beech fructification and bank vole abundance on human PUUV incidence were statistically analysed by a GLMM (generalized linear mixed model; level of significance α < 5%) with binomial error distribution and a logit link function using R software [44]. As human PUUV incidences are proportion data, a proportional response variable (2-vector variable) was generated from the number of reported human PUUV cases and the difference between the number of inhabitants and the number of reported human PUUV cases (= number of uninfected people). In the GLMM beech fructification and bank vole abundance were the covariates. Additionally, a fixed effect of the proportion of beech forest per state was included in the analysis, because human infection risk with PUUV may also depend on the occurrence of suitable habitat (beech forests) for PUUV rodent hosts. Prior to this, the effect of beech fructification on bank vole abundance was analyzed by a GLMM (level of significance α < 5%) with binomial error distributions and a logit link function, which was also used to test for collinearity between these two parameters. Bank vole abundance (2-vector variable generated from trap success and trap failure per 100 trap nights) was the dependent variable and beech fructification the covariate.

In each model Federal State was included as a random factor, because the analysis included averaged data of 7 German states (Bavaria, Brandenburg, Hesse, Lower Saxony, Mecklenburg-Western Pomerania, Saxony and Thuringia) resulting in a sample size of N = 63. Best models were selected based on the Akaike information criterion (AIC). For each model a pseudo-R^2^ for GLMMs (R^2^_conditional_ = variance explained by fixed and random factors) was estimated using the function ‘r.squaredGLMM’ from the ‘MuMIn’-package [45, 46].

## Results

### Beech fructification

**Fig 1:**
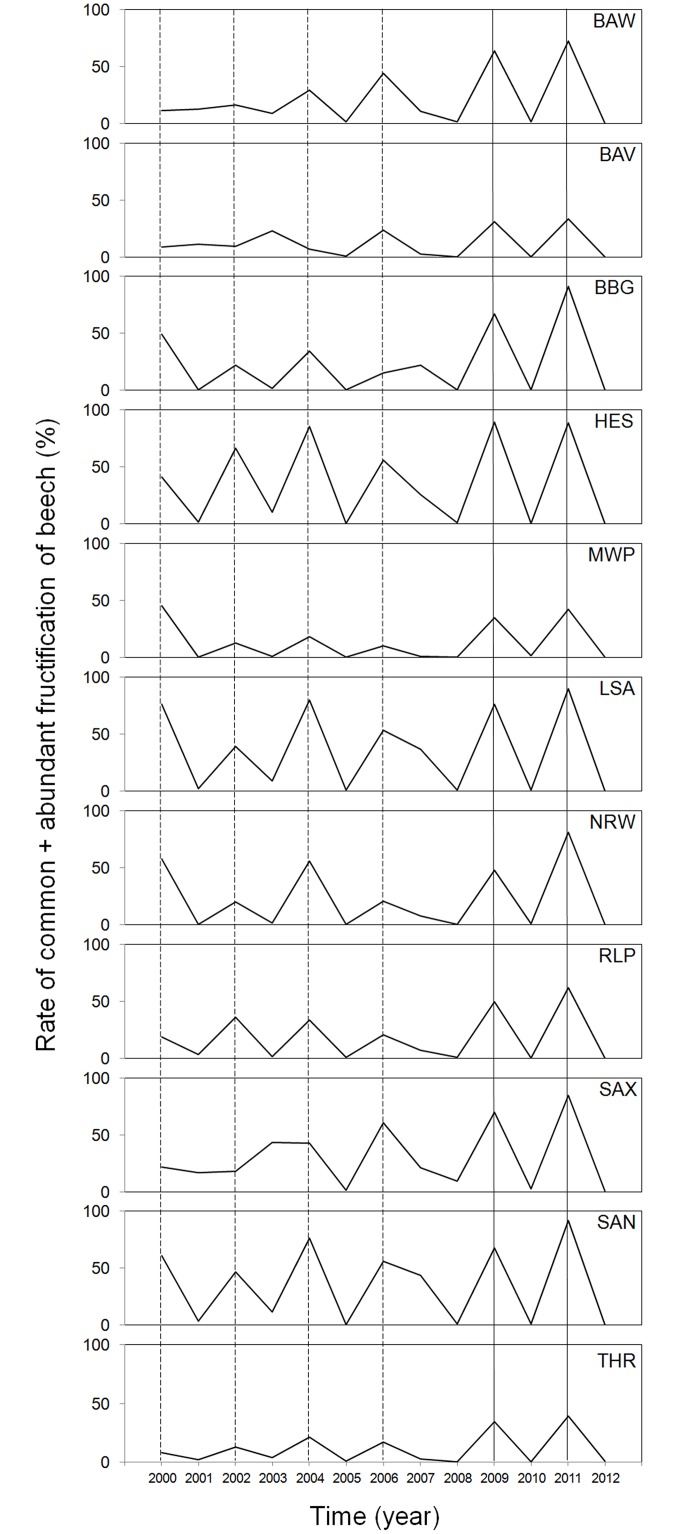
Time series of beech fructification intensity in 11 Federal States of Germany. BAW = Baden-Wuerttemberg, BAV = Bavaria, BBG = Brandenburg, HES = Hesse, MWP = Mecklenburg-Western Pomerania, LSA = Lower Saxony, NRW = North Rhine-Westphalia, RLP = Rhineland-Palatinate, SAX = Saxony, SAN = Saxony-Anhalt, THR = Thuringia 2000–2012. Beech masts synchronous in all Federal States are highlighted by vertical solid lines and beech masts synchronous in 4–7 of 11 of Federal States are indicated by vertical dashed lines.

The temporal pattern of beech fructification changed in 2008 from a mix of 2–3 year frequency of masting depending on Federal State to a highly synchronized 2-year cycle across all German Federal States (Fig 1). Prior to 2006, beech mast in three Federal States (Saxony, Bavaria, Baden-Wuerttemberg) was at least partially off cycle and beech fructification in that period was generally minor (no mast) in some Federal States such as Bavaria and Thuringia. After 2008 beech mast was obvious and absolutely synchronous in 2009 and 2011 for all states with a fructification rate of > 30%. Further, mean beech fructification intensity in mast years in Germany increased from < 50% in 2000 (36±24%), 2002 (27±18%), 2004 (44±27%), and 2006 (34±20%) to > 50% in 2009 (58±19%) and 2011 (71±23%).

### Bank vole abundance

The GLMM demonstrated that bank vole abundance was significantly affected by preceding beech fructification (z = 9.13, *p* < 0.001; Table 3), which was concordant with our hypothesis, and 33% (R^2^_conditional_) of the variance in bank vole abundance were explained.

**Table 3:** Model results of the correlations between beech fructification, bank vole abundance and human PUUV incidence. Parameter coefficients of generalized linear mixed models with binomial error distribution used to examine the influence of beech fructification on bank vole abundance and of beech fructification and bank vole abundance on human PUUV incidence.

Dependent Variable	Parameter	Estimate	(SE)	z	*p*	
Bank vole abundance	Intercept	-3.98	(0.41)	-9.61	***<0*.*001***	***
	Beech fructification	2.53	(0.28)	9.13	***<0*.*001***	***
Human PUUV incidence	Intercept	-15.41	(0.83)	-18.47	***<0*.*001***	***
	Beech fructification	4.38	(0.17)	25.18	***<0*.*001***	***
	Bank vole abundance	0.02	(0.01)	2.51	***0*.*012***	*

**Fig 2:**
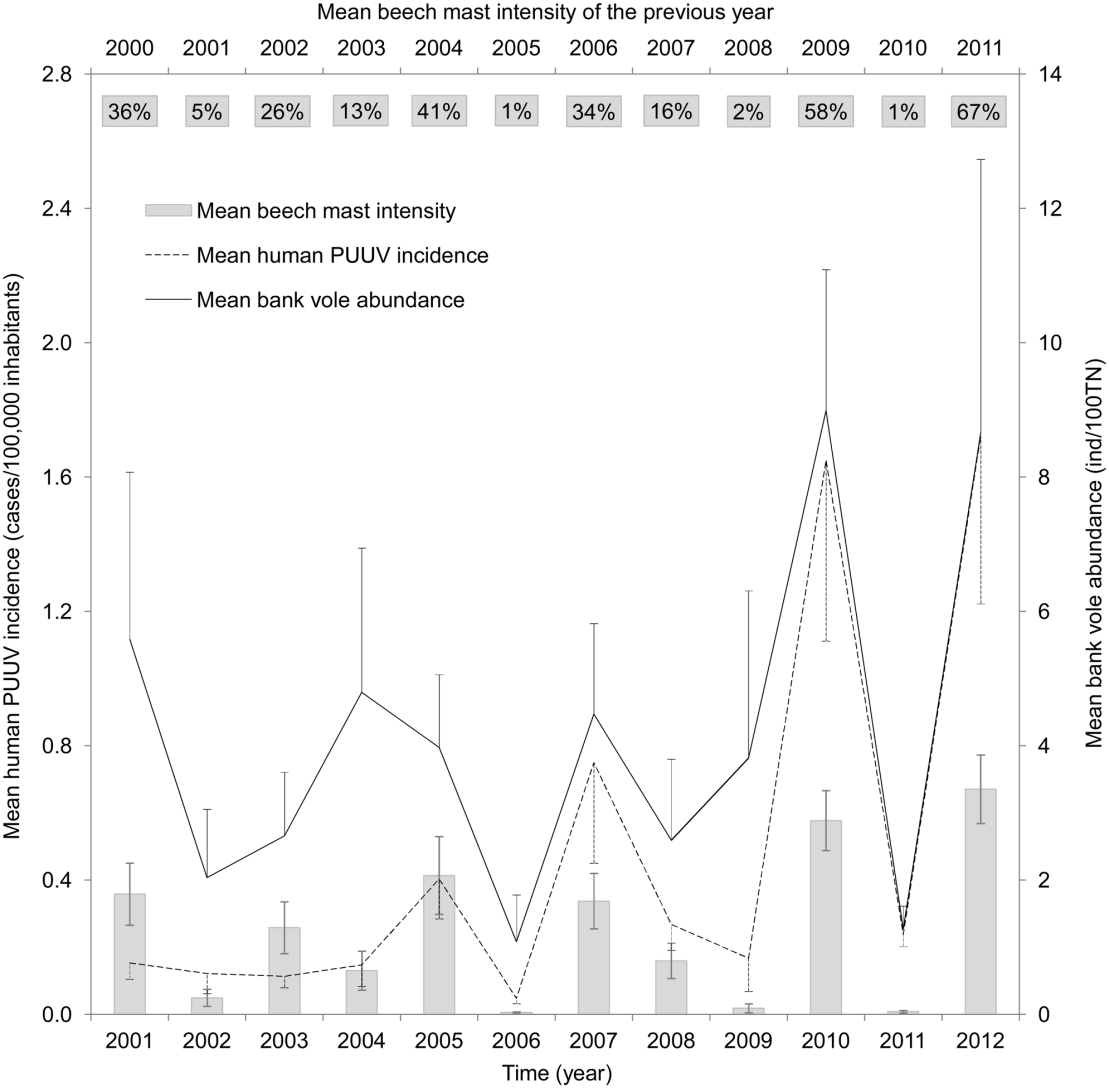
Mean beech mast intensity of the previous year, bank vole abundance, and human PUUV incidence in Germany 2001–2012. Bars = mean beech mast intensity of the previous year, solid line = bank vole abundance, and broken line = human PUUV incidence. Values are mean values ± one standard error from 7 Federal States. Upper bold values are beech mast intensity corresponding to bars at the bottom. (PUUV = Puumala virus; TN = trap nights).

According to trap success, bank vole abundance varied considerably between states. On average, the highest bank voles abundances were found in Lower Saxony in 2010 and 2012 (17–18 ind/100TN), which corresponds to the second highest (2009) and highest (2011) beech mast in the respective previous years (76–90%) (Fig 1). On average, bank vole abundance maxima occurred in 2001, 2004 and 2005, 2007, 2010, and 2012 (Fig 2). High bank vole abundance was preceded by beech mast in the previous year, except in 2004. In 2003–2004 there seemed to be an inverse pattern as increasing bank vole abundance and low beech fructification in the previous year were recorded.

### Human PUUV infections

**Fig 3:**
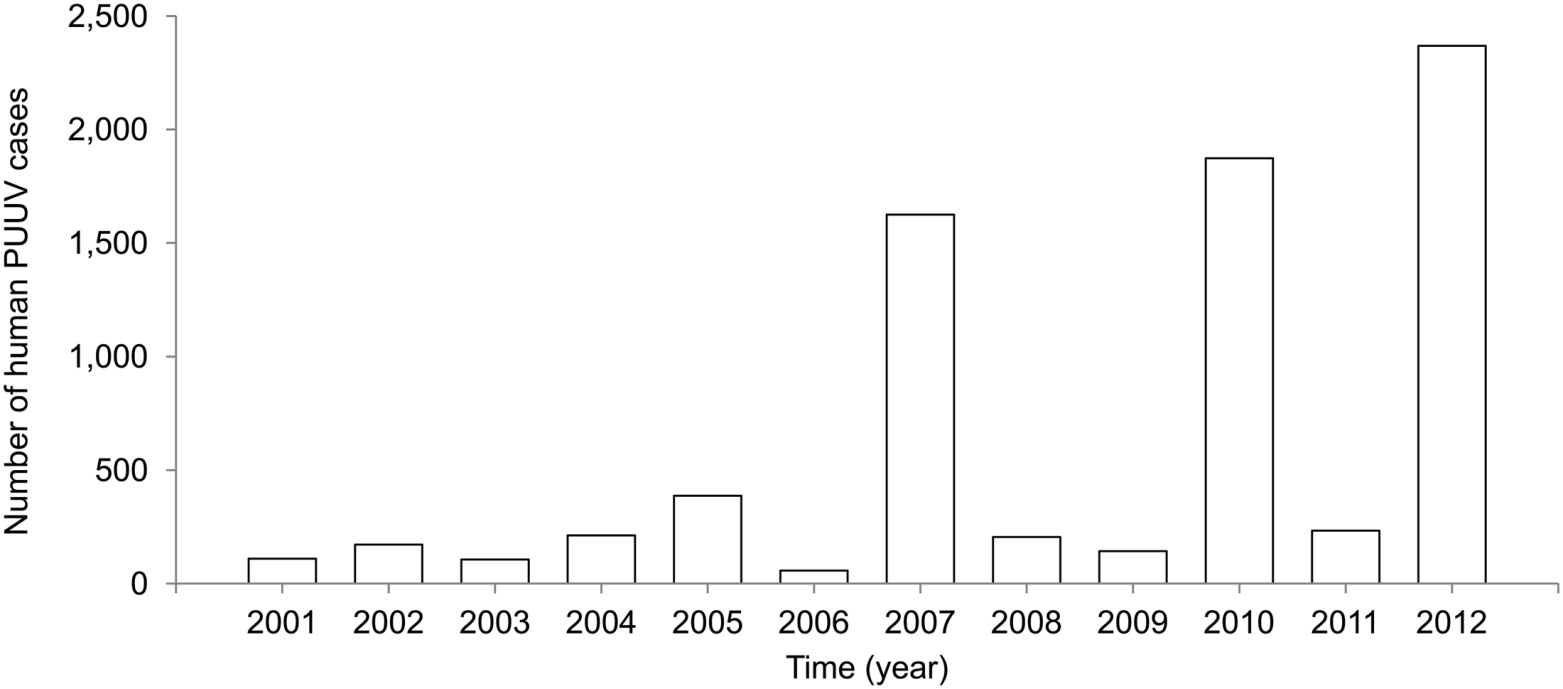
Total reported annual human Puumala virus (PUUV) cases in Germany 2001–2012 (all 16 Federal States). Source: Robert Koch-Institute, SurvStat, http://www3.rki.de/SurvStat, data status: 06/27/2014.

After the obligation to notify hantavirus infections (mostly PUUV) was instigated in Germany in 2001, three major NE outbreaks occurred in 2007, 2010, and 2012 (Fig 3). In these PUUV outbreak years, the number of annually diagnosed and reported cases of human PUUV infections steadily increased from 1,625 (2007) to 1,873 (2010) and 2,369 (2012) (Table 2). In the years between these outbreak years, 57–387 cases were reported. In contrast, during the first few years after the obligation to notify hantavirus infections was instigated, there was no obvious rise of notified cases whereas bank vole abundance and beech fructification should have promoted increased human infection (Fig 2). Human PUUV infections since 2001 (7,589 cases) were most frequent in southern Germany (with three quarters of all cases in Baden-Wuerttemberg and Bavaria, Table 2), followed by 17% of cases in West and North-West Germany (North Rhine-Westphalia and Lower Saxony), and 5% in Central Germany (Hesse). Less than 1% of cases were reported from East Germany (Table 2).

### Influence of beech mast and bank vole dynamics on human PUUV infections

The correlation between bank vole abundance and beech fructification was low (R^2^_conditional_ = 0.33). Therefore, collinearity among these two predictors was not considered an issue and both parameters were included in the GLMM to analyse their combined effect on human PUUV incidence. We ran two model versions including and excluding the proportion of beech forest per state. Because there was no effect of the proportion of beech forest per state on human PUUV incidence (*p = 0*.*138*) and Federal State was included as a random factor in the model, we decided to drop that parameter in further analyses.

Beech fructification positively affected human PUUV incidence in the following year (*z* = 25.18, *p* < 0.001) and so did bank vole abundance (*z* = 2.51, *p* = 0.012) (Table 3). The model explained 65% (R^2^_conditional_) of the variance in human PUUV incidence.

All but one peak in human PUUV incidence (2005, 2007, 2010, and 2012) were preceded by beech mast in the previous year and accompanied by an increase of bank vole abundance (Fig 2).

## Discussion

We showed that masting of common beech occurred prior to years with high human PUUV incidence and that these incidence peaks were regularly accompanied by increased bank vole population densities. For the first time, we could establish a direct relation between these three parameters involved in human PUUV epidemics investigating well replicated time series of up to 12 years length and on a large spatial scale. The combined analysis of the impact of beech fructification and bank vole abundance as predictors of human PUUV incidence improved the degree of explanation of human PUUV incidence in the central European bank vole—PUUV system.

Our results correspond to findings of an earlier, however regional study, which was conducted in Baden-Wuerttemberg, Germany. Piechotowski, Brockmann [40] showed that human hantavirus infections were affected by climatic conditions, which they supposed to be linked to food availability and rodent host dynamics. The results of our study show that food availability via beech fructification indeed seems to drive bank vole abundance and that both beech fructification and bank vole abundance seem to drive human PUUV incidence. At such regional scale several environmental parameters including food supply, beech forest cover and weather may influence human NE incidence [47]. Beech mast as food supply to bank voles, and population abundance of the rodent host considered in our study matter well beyond the regional scale and seem to significantly determine temporal infection patterns of PUUV infection in humans.

As hypothesised, we found a clear positive correlation between rodent host species abundance and human infections with PUUV. Although the occurrence of the reservoir species bank vole is essential for the transmission of PUUV to humans, the human infection risk is not simply linked to rodent host abundance but to the abundance of infected rodent hosts. The ocurrence of PUUV in host populations seems to be highly patchy [48]. Bennett, Clement [49], for example, report that in Great Britain there are PUUV incidences neither in humans nor in animals, although bank voles are common. Because of the absence of PUUV from some vole populations in Germany human incidences in the corresponding Federal States are very low (Eastern and Northern Germany). This may partially explain why not all variance in human PUUV incidence was explained by bank vole abundance and beech fructification alone. However, the model performed exceptionally well given the complexity of disease transmission involving many other factors potentially affecting human PUUV incidence, which are considered below.

The highest bank vole abundance averaged per year was found in Lower Saxony (North-western Germany) in 2010 and 2012, but the number of human infections was only the fourth highest after Baden-Wuerttemberg, Bavaria, and North Rhine-Westphalia (Table 2). This could have been an effect of data recording for human incidence, which is discussed below. Nevertheless, bank vole abundance seems to be a useful predictor for NE outbreaks in Finland even without information about the occurrence or dynamics of the hantavirus in the host population [50]. Probably, PUUV is more regularly distributed over the entire bank vole population in Finland, compared to patchy distribution in Germany.

The study results confirmed our hypotheses that beech fructification is indirectly affecting human PUUV incidence and that it has a direct impact on bank vole abundance. The latter is supported by the finding that bank vole abundance can be predicted by specific constellations of weather parameters that are associated to beech masting [51]. Beech seeds are a main food resource for forest dwelling rodents. High tree seed production (masting) provides best food conditions for rodents causing a better over-winter survival or even winter breeding and an early start of the breeding season in the following year [30, 37, 52–54]. The increase in population density of the reservoir species bank vole has a great influence on the transmission of the PUUV within and among reservoir populations [4, 5, 55] and hence on the amount of shed virus particles to the environment, which are responsible for human infections. This mechanism may cause the strong but rather indirect correlation we found between beech fructification and human PUUV infections.

Apart from rodent host density and tree mast also human exposure and behavior can affect human infection risk [56]. High risk groups include forest workers [57], but also residency in rural areas with nearby forests matters [58]. Clement and Van der Groen [24] indicated that dense forest habitats harbour rodent populations with higher PUUV seroprevalence compared to sparsely forested parts and therefore have a major impact on hantavirus disease transmission. Such habitat factors also seem to influence the risk of infection with PUUV for humans. Further, behavior of the rodent host itself due to population density and climatic conditions can impact on transmission processes within host populations and therefore affect human infection risk [59].

The correlations of weather, beech mast, bank vole dynamics and human PUUV infection rate may allow to predict human PUUV infection risk well in advance. This would provide sufficient time for authorities to issue public warnings and to initiate safety precaution for high risk groups such as forestry workers, military personnel and field biologists.

In the 1990s, beech mast in Germany occurred at a three-year-cycle (Federal Forest Status Reports, BMEL (German Federal Ministry of Food and Agriculture)), but since 1998, it changed into a 2–3 year cycle. This is similar to Southern Sweden where beech mast shifted from a ~5 year cycle since the end of the seventeenth century to an interval of 2.5 years since 1974 [60]. In contrast to Belgium, where beech mast does not occur synchronously on regional scale [41], beech mast in Germany was spatially synchronized since 2001 (Fig 1). The frequent and synchronized beech mast in Germany—and possibly other parts of the Central European beech population—indicates increased risk for bank vole outbreaks and associated human PUUV infections. Within state variation of beech mast was not considered because no information beyond state scale was available. Beech mast is driven mainly by climate [60, 61]. Ongoing climate change is suggested to increase the number of years with high bank vole abundances by the end of this century [51] despite recent dampening of small rodent cycle amplitudes in many parts of Europe [62]. According to that and because of the importance of climatic parameters like summer temperature and precipitation in the previous year for fruiting of beech trees [60, 61, 63], climate change may also explain the recent large scale increase in beech masting.

Apparently, tree seed production and rodent host abundance seem to be useful predictors for human PUUV infection outbreaks [35, 50]. Surprisingly, we found that beech mast has a stronger effect on human PUUV infections than bank vole dynamics. There might have been an effect of coarse temporal and spatial scale for data collection. PUUV infections were recorded according to residence of the patient (not the location of infection), according to the date NE was diagnosed (not the date of infection), and infections often remain undetected [27]. Moreover, bank vole trapping was conducted for plant protection purpose (debarking of young trees), so it is likely that trap success was not always measured in old-growth forests where beech mast occurs at highest levels and where it was monitored. In this scenario bank voles considered for abundance measurements would not always have been exposed to the full effect of beech mast, which may also contribute to the higher effect of beech mast compared to bank vole abundance on human PUUV incidence.

In conclusion, our study demonstrated that Germany-wide human PUUV infections are highly correlated to bank vole abundance in the present year, as well as beech fructification in the previous year. So increased numbers of human PUUV infections are not just a consequence of increased awareness towards the disease. We demonstrate that the effect of beech fructification on human PUUV infections is indirect but seemed to be more pronounced than the effect of rodent host abundance. The mast-rodent-human-PUUV system is complex and affected by many factors interacting such as climate, human and vole behavior. The close relations between beech mast, rodent population dynamics, and human PUUV infections we have found in this study can further the development of predictive models for bank vole population dynamics and the related infection risk for humans with the PUUV. Such models can be used for human health protection as well as for plant protection.

## References

[1] Jones KE, Patel NG, Levy MA, Storeygard A, Balk D, Gittleman JL, et al Global trends in emerging infectious diseases. Nature. 2008;451(7181):990–3. 10.1038/nature06536 18288193 PMC5960580

[2] Mills JN. Biodiversity loss and emerging infectious disease: an example from the rodent-borne hemorrhagic fevers. Biodiversity. 2006;7(1):9–17. 10.1080/14888386.2006.9712789

[3] Lloyd-Smith JO, George D, Pepin KM, Pitzer VE, Pulliam JRC, Dobson AP, et al Epidemic dynamics at the human-animal interface. Science. 2009;326(5958):1362–7. 10.1126/science.1177345 19965751 PMC3891603

[4] Mills JN, Ksiazek TG, Peters CJ, Childs JE. Long-term studies of hantavirus reservoir populations in the southwestern United States: a synthesis. Emerg Infect Dis. 1999;5(1):135–42. 10081681 10.3201/eid0501.990116 PMC2627702

[5] Escutenaire S, Chalon P, Verhagen R, Heyman P, Thomas I, Karelle-Bui L, et al Spatial and temporal dynamics of Puumala hantavirus infection in red bank vole (Clethrionomys glareolus) populations in Belgium. Virus Res. 2000;67(1):91–107. 10.1016/S0168-1702(00)00136-2 10773322

[6] Killilea ME, Swei A, Lane RS, Briggs CJ, Ostfeld RS. Spatial dynamics of Lyme disease: a review. Ecohealth. 2008;5(2):167–95. 10.1007/s10393-008-0171-3 18787920

[7] Ostfeld RS, Canham CD, Oggenfuss K, Winchcombe RJ, Keesing F. Climate, deer, rodents, and acorns as determinants of variation in Lyme-disease risk. PloS Biol. 2006;4(6):1058–68. 10.1371/journal.pbio.0040145 PMC1457019 16669698

[8] Schauber EM, Ostfeld RS, Evans J, A. S. What is the best predictor of annual Lyme disease incidence: weather, mice, or acorns? Ecol Appl. 2005;15(2):575–86. 10.1890/03-5370

[9] Jacob J, Tkadlec E. Rodent outbreaks in Europe: dynamics and damage In: Singleton GR, Belmain S, Brown PR, Hardy B, editors. Rodent outbreaks—Ecology and impacts. Los Baños, Philippines: International Rice Research Institute; 2010 p. 207–23.

[10] Davis S, Calvet E, Leirs H. Fluctuating rodent populations and risk to humans from rodent-borne zoonoses. Vector Borne Zoonotic Dis. 2005;5(4):305–14. 10.1089/vbz.2005.5.305 16417426

[11] Vaheri A, Henttonen H, Voutilainen L, Mustonen J, Sironen T, Vapalahti O. Hantavirus infections in Europe and their impact on public health. Rev Med Virol. 2013;23(1):35–49. 10.1002/rmv.1722 22761056

[12] Schmaljohn C, Hjelle B. Hantaviruses: A global disease problem. Emerg Infect Dis. 1997;3(2):95–104. 9204290 10.3201/eid0302.970202 PMC2627612

[13] Kang HJ, Bennett SN, Sumibcay L, Arai S, Hope AG, Mocz G, et al Evolutionary insights from a genetically divergent hantavirus harbored by the European common mole (*Talpa europaea*). PloS One. 2009;4(7). 10.1371/journal.pone.0006149 PMC2702001 19582155

[14] Schlegel M, Radosa L, Rosenfeld UM, Schmidt S, Triebenbacher C, Lohr PW, et al Broad geographical distribution and high genetic diversity of shrew-borne Seewis hantavirus in Central Europe. Virus Genes. 2012;45(1):48–55. 10.1007/s11262-012-0736-7 22467179

[15] Sumibcay L, Kadjo B, Gu SH, Kang HJ, Lim BK, Cook JA, et al Divergent lineage of a novel hantavirus in the banana pipistrelle (*Neoromicia nanus*) in Cote d'Ivoire. Virol J. 2012;9:34 10.1186/1743-422x-9-34 22281072 PMC3331809

[16] Brummer-Korvenkontio M, Vaheri A, Hovi T, Von Bonsdorff CH, Vuorimies J, Manni T, et al Nephropathia epidemica: detection of antigen in bank voles and serologic diagnosis of human infection. J Infect Dis. 1980;141(2):131–4. 6102587 10.1093/infdis/141.2.131

[17] Makary P, Kanerva M, Ollgren J, Virtanen MJ, Vapalahti O, Lyytikäinen O. Disease burden of Puumala virus infections, 1995–2008. Epidemiol Infect. 2010;138(10):1484–92. 10.1017/S0950268810000087 20109263

[18] Schmaljohn CS, Hasty SE, Dalrymple JM, Leduc JW, Lee HW, Vonbonsdorff CH, et al Antigenic and genetic properties of viruses linked to hemorrhagic-fever with renal syndrome. Science. 1985;227(4690):1041–4. 10.1126/science.2858126 2858126

[19] Meyer BJ, Schmaljohn CS. Persistent hantavirus infections: characteristics and mechanisms. Trends Microbiol. 2000;8(2):61–7. 10.1016/S0966-842x(99)01658-3 10664598

[20] Kallio ER, Poikonen A, Vaheri A, Vapalahti O, Henttonen H, Koskela E, et al Maternal antibodies postpone hantavirus infection and enhance individual breeding success. Proc Biol Sci. 2006;273(1602):2771–6. 10.1098/rspb.2006.3645 17015326 PMC1635497

[21] Kallio ER, Voutilainen L, Vapalahti O, Vaheri A, Henttonen H, Koskela E, et al Endemic hantavirus infection impairs the winter survival of its rodent host. Ecology. 2007;88(8):1911–6. 10.1890/06-1620.1 17824420

[22] Faulde M, Sobe D, Kimmig P, Scharninghausen J. Renal failure and hantavirus infection in Europe. Nephrol Dial Transpl. 2000;15(6):751–3. 10.1093/Ndt/15.6.751 10831620

[23] Vapalahti O, Mustonen J, Lundkvist A, Henttonen H, Plyusnin A, Vaheri A. Hantavirus infections in Europe. Lancet Infect Dis. 2003;3(10):653–61. 10.1016/S1473-3099(03)00774-6 14522264

[24] Clement J, Van der Groen G. Acute hantavirus nephropathy in Belgium: preliminary results of a sero-epidemiological study. Adv Exp Med Biol. 1987;212:251–63. 10.1007/978-1-4684-8240-9_32 2887093

[25] Zeier M, Andrassy K, Waldherr R, Ritz E. Acute renal failure due to Hantaan virus—a case-report from the Federal Republic of Germany. Dtsch Med Wochenschr. 1986;111(6):207–10. 3080305 10.1055/s-2008-1068427

[26] Clement J, Underwood P, Ward D, Pilaski J, LeDuc J. Hantavirus outbreak during military manoeuvres in Germany. Lancet. 1996;347(8997):336 10.1016/S0140-6736(96)90519-X 8569401

[27] Clement J, Maes P, de Strihou CV, van der Groen G, Barrios JM, Verstraeten WW, et al Beechnuts and outbreaks of nephropathia epidemica (NE): of mast, mice and men. Nephrol Dial Transpl. 2010;25(6):1740–6. 10.1093/ndt/gfq122 20237057

[28] Hanski I, Hansson L, Henttonen H. Specialist predators, generalist predators, and the microtine rodent cycle. J Anim Ecol. 1991;60:353–67.

[29] Selas V. Explaining bank vole cycles in southern Norway 1980–2004 from bilberry reports 1932–1977 and climate. Oecologia. 2006;147(4):625–31. 10.1007/s00442-005-0326-7 16344969

[30] Pucek Z, Jedrzejewski W, Jedrzejewska B, Pucek M. Rodent population dynamics in a primeval deciduous forest (Bialowieza National Park) in relation to weather, seed crop, and predation. Acta Theriol. 1993;38:199–232.

[31] Hansson L, Jedrzejewska B, Jedrzejewski W. Regrional differences in dynamics of bank vole populations in Europe. Pol J Ecol. 2000;48:163–77.

[32] Viro P, Sulkava S. Food of the bank vole in northern Finnish spruce forests. Acta Theriol. 1985;30(9–20):259–66.

[33] Jensen TS. Seed-seed predator interactions of European beech, *Fagus sylvatica* and forest rodents, *Clethrionomys glareolus* and *Apodemus flavicollis* . Oikos. 1985;44:149–56. 10.2307/3544056

[34] Schmitz F, Polley H, Hennig P, Schwitzgebel F, Kriebitzsch WU. Die zweite Bundeswaldinventur—BWI2: Das Wichtigste in Kürze. Bonn: Bundesministerium für Verbraucherschutz, Ernährung und Landwirtschaft; 2004.

[35] Kallio ER, Begon M, Henttonen H, Koskela E, Mappes T, Vaheri A, et al Cyclic hantavirus epidemics in humans: predicted by rodent host dynamics. Epidemics. 2009;1:101–7. 10.1016/j.epidem.2009.03.002 21352757

[36] Olsson GE, Dalerum F, Hörnfeldt B, Elgh F, Palo TR, Juto P, et al Human hantavirus infections, Sweden. Emerg Infect Dis. 2003;9(11):1395 10.3201/eid0911.030275 14718081 PMC3035548

[37] Clement J, Vercauteren J, Verstraeten WW, Ducoffre G, Barrios JM, Vandamme A-M, et al Relating increasing hantavirus incidences to the changing climate: the mast connection. Int J Health Geogr. 2009;8(1):1–11. 10.1186/1476-072X-8-1 19149870 PMC2642778

[38] Tersago K, Verhagen R, Servais A, Heyman P, Ducoffre G, Leirs H. Hantavirus disease (nephropathia epidemica) in Belgium: effects of tree seed production and climate. Epidemiol Infect. 2008;137(02):250–6. 10.1017/S0950268808000940 18606026

[39] Niklasson B, Hornfeldt B, Lundkvist A, Bjorsten S, Leduc J. Temporal dynamics of Puumala virus antibody prevalence in voles and of nephropathia epidemica incidence in humans. Am J Trop Med Hyg. 1995;53(2):134–40. 7677213 10.4269/ajtmh.1995.53.134

[40] Piechotowski I, Brockmann SO, Schwarz C, Winter CH, Ranft U, Pfaff G. Emergence of hantavirus in South Germany: rodents, climate and human infections. Parasitol Res. 2008;103:131–7. 10.1007/s00436-008-1055-8 19030895

[41] Heyman P, Thoma BR, Marie JL, Cochez C, Essbauer SS. In search for factors that drive hantavirus epidemics. Front Physiol. 2012;3:237 10.3389/fphys.2012.00237 22934002 PMC3429022

[42] Jensen TS. Seed production and outbreaks of non-cyclic rodent populations in deciduous forests. Oecologia. 1982;54:184–92. 10.1007/BF00378391 28311427

[43] Eichhorn J, Roskams P, Ferretti M, Mues V, Szepesi A, Durrant D. Manual Part IV: Visual assessment of crown condition and damaging agents. 49 pp. In: Manual on methods and criteria for harmonized sampling, assessment, monitoring and analysis of the effects of air pollution on forests. Hamburg, [http://www.icp-forests.org/Manual.htm]: UNECE ICP Forests Programme Co-ordinating Centre; 2010.

[44] R Development Core Team. R: A language and environment for statistical computing. Version 3.03 ed. Vienna, Austria Available: http://www.R-project.org: R Foundation for Statistical Computing; 2014.

[45] Nakagawa S, Schielzeth H. A general and simple method for obtaining R2 from generalized linear mixed-effects models. Methods Ecol Evol. 2013;4(2):133–42. 10.1111/j.2041-210x.2012.00261.x

[46] Johnson PC. Extension of Nakagawa & Schielzeth's R2GLMM to random slopes models. Methods Ecol Evol. 2014 10.1111/2041-210X.12225 PMC4368045 25810896

[47] Schwarz AC, Ranft U, Piechotowski I, Childs JE, Brockmann SO. Risk factors for human infection with Puumala virus, Southwestern Germany. Emerg Infect Dis. 2009;15(7):1032–9. 10.3201/eid1507.081413 19624917 PMC2744254

[48] Schlegel M, Jacob J, Krüger DH, Rang A, Ulrich RG. Hantavirus emergence in rodents, insectivores and bats: what comes next? In: Johnson N, editor. The Role of Animals in Emerging Viral Diseases. Boston: Academic Press; 2014 p. 235–92.

[49] Bennett E, Clement J, Sansom P, Hall I, Leach S, Medlock J. Environmental and ecological potential for enzootic cycles of Puumala hantavirus in Great Britain. Epidemiol Infect. 2010;138(1):91–8. 10.1017/S095026880999029X 19563697

[50] Amirpour Haredasht S, Taylor CJ, Maes P, Verstraeten WW, Clement J, Barrios M, et al Model-based prediction of nephropathia epidemica outbreaks based on climatological and vegetation data and bank vole population dynamics. Zoonoses Public Health. 2013;60(7):461–77. 10.1111/Zph.12021 23176630

[51] Imholt C, Reil D, Eccard JA, Jacob D, Hempelmann N, Jacob J. Quantifying the past and future impact of climate on outbreak patterns of bank voles (*Myodes glareolus*). Pest Manag Sci. 2014 10.1002/ps.3838 24889216

[52] Andrzejewski R. Supplementary food and the winter dynamics of bank vole populations. Acta Theriol. 1975;20(2):23–40.

[53] Eccard JA, Ylönen H. Initiation of breeding after winter in bank voles: effects of food and population density. Can J Zool. 2001;79(10):17431753 10.1139/z01-133

[54] Verhagen R, Leirs H, Verheyen W. Demography of *Clethrionomys glareolus* in Belgium. Pol J Ecol. 2000;48:113–23.

[55] Sauvage F, Langlais M, Pontier D. Predicting the emergence of human hantavirus disease using a combination of viral dynamics and rodent demographic patterns. Epidemiol Infect. 2007;135(01):46–56. 10.1017/S0950268806006595 16753079 PMC2870550

[56] Crowcroft NS, Infuso A, Ilef D, Le Guenno B, Desenclos JC, Van Loock F, et al Risk factors for human hantavirus infection: Franco-Belgian collaborative case-control study during 1995–6 epidemic. Br Med J. 1999;318(7200):1737–8. 10.1136/bmj.318.7200.1737 10381709 PMC31102

[57] Mertens M, Hofmann J, Petraityte-Burneikiene R, Ziller M, Sasnauskas K, Friedrich R, et al Seroprevalence study in forestry workers of a non-endemic region in eastern Germany reveals infections by Tula and Dobrava-Belgrade hantaviruses. Med Microbiol Immunol. 2011;200(4):263–8. 10.1007/s00430-011-0203-4 21611907

[58] Abu Sin M, Stark K, van Treeck U, Dieckmann H, Uphoff H, Hautmann W, et al Risk factors for hantavirus infection in Germany, 2005. Emerg Infect Dis. 2007;13(9):1364–6. 18252110 10.3201/eid1309.070552 PMC2857305

[59] Khalil H, Hornfeldt B, Evander M, Magnusson M, Olsson G, Ecke F. Dynamics and drivers of Hantavirus prevalence in rodent populations. Vector Borne Zoonotic Dis. 2014;14(8):537–51. 10.1089/vbz.2013.1562 25072983

[60] Övergaard R, Gemmel P, Karlsson M. Effects of weather conditions on mast year frequency in beech (*Fagus sylvatica* L.) in Sweden. Forestry. 2007;80(5):553–63. 10.1093/forestry/cpm020

[61] Piovesan G, Adams JM. Masting behaviour in beech: linking reproduction and climatic variation. Can J Bot. 2001;79(9):1039–47. 10.1139/b01-089

[62] Cornulier T, Yoccoz NG, Bretagnolle V, Brommer JE, Butet A, Ecke F, et al Europe-wide dampening of population cycles in keystone herbivores. Science. 2013;340(6128):63–6. 10.1126/science.1228992 23559246

[63] Schmidt W. Temporal variation in beech masting (*Fagus sylvatica* L.) in a limestone beech forest (1981–2004). Allg Forst Jagdztg. 2006;177(1):9–19.

